# Active Reduction of Apparent Cable Capacitance

**DOI:** 10.3390/s23198319

**Published:** 2023-10-08

**Authors:** Alexander Melin, Michael Roberts, Roger Kisner

**Affiliations:** 1 Oak Ridge National Laboratory, Oak Ridge, TN 37831, USA; 2 Department of Electrical Engineering and Computer Science, University of Tennessee, Knoxville, TN 37996, USA; mjr@utk.edu; 3 Valtok, Karns, TN 37931, USA; roger@valtok.com

**Keywords:** coaxial cable, triaxial cable, capacitance reduction, bandwidth

## Abstract

This paper presents a method for reducing apparent cable capacitance seen by sensors. The proposed method is designed for sensing in extreme environments including ultra-high temperatures, but can be applied in benign environments as well. By reducing the cable capacitance, high speed signals can be transmitted over longer distances, allowing the application of advanced control systems that require high bandwidth data for stable operation. A triaxial cable with an associated guard circuit is developed that actively reduces cable capacitance while also rejecting extraneous electric and magnetic interference on the signal. The active capacitance reduction method developed is tested experimentally and shown to reduce apparent cable capacitance to two percent of the static value.

## 1. Introduction

The extreme environments encountered in aerospace, fusion energy, fission energy, and concentrated solar energy have severe restrictions on materials that can be used in sensors.

Some applications for extreme environment sensors are high temperature magnetic bearings developed by the National Aeronautical and Space Administration (NASA) [1] and magnetically levitated molten salt pumps being developed by the Department of Energy (DOE) for molten salt reactors (MSRs), fusion energy, and concentrated solar energy [2,3]. In both applications, it is required to dynamically measure the location of a rotor at high sample rates to stabilize the magnetic bearings using feedback control.

In the case of the DOE molten salt pump, the design removes the need for rotating seals and roller element bearings, which are components with a high failure rate. A major challenge in this design is the requirement for the rotor position sensors to be in close physical proximity to the rotor. In this application, temperatures can reach 800 °C, well above the Curie temperature of most materials. In addition, the salt chemistry is highly corrosive, which requires using corrosion barriers to protect materials that are not salt compatible [2,3].

The cabling connecting the sensors in the extreme environment to the signal processing and data acquisition also has similar design challenges. In addition, the electronic components need to be in a relatively benign environment, which is often far from the sensor. Components like magnetic bearings require high frequency position measurements for stable operation. As the cable length increases, the apparent capacitance (as seen by the sensor) also increases. The RS-232-C standard limits cable lengths to 50 m or below 2500 pF [4]. In [5], the empirically determined maximum lengths for RS-232 cables sending data a 19.2 kilobaud are about ten times the RS-232-C standard. High performance mainframe cables developed by IBM support 330 Mb/s data transmission rates at cable lengths up to 20 m [6]. The maximum cable length for contactor control cables is calculated in [7]. In all the preceding references, a common theme is that cable capacitance limits performance. Increasing cable capacitance reduces the overall sensor bandwidth, reduces data transmission rates, and decreases the signal-to-noise ratio. The need for reducing the apparent cable capacitance for stable high temperature magnetic bearing control motivates this work.

**Figure 1:**
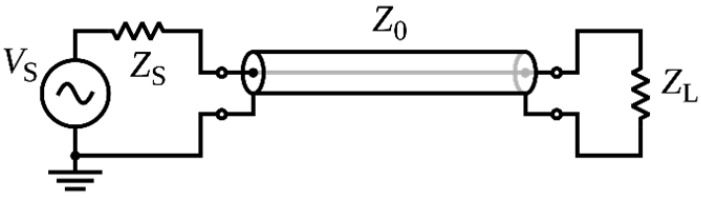
Typical implementation of a coaxial cable with signal return path through the shield.

In addition to low capacitance, the cables need to shield against electromagnetic interference from the electric motor providing torque for the rotor, pulse width modulated magnetic bearing drive signals, along with magnetic fields from both motor and magnetic bearings. Coaxial cables shield the signal from extraneous electric and magnetic fields, because it is imposed on the center conductor and shield as common mode noise, which is then rejected by the differential amplifier used to increase the sensor signal amplitude. Figure 1 shows a typical implementation of a coaxial cable. $Z_{L}$ represents the sensor which is driven by the voltage $V_{S}$.

**Figure 2:**
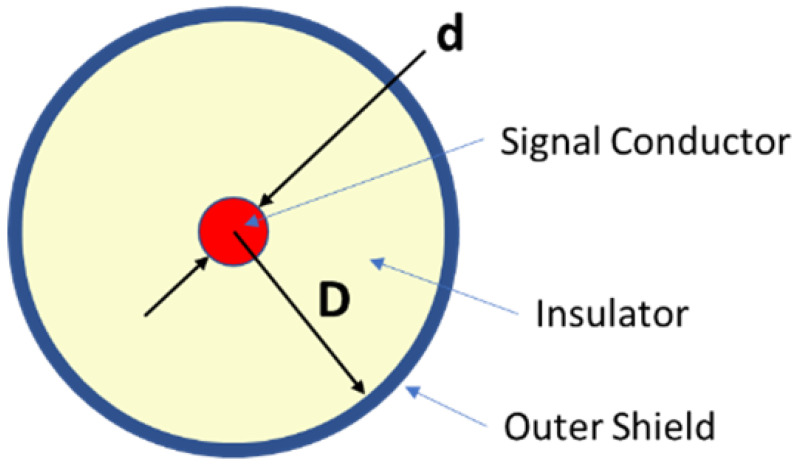
Cross section representation of a coaxial cable.

The internal capacitance of coaxial cable geometry shown in Figure 2 is given by

(1) $$\begin{array}{c} \frac{C}{l}=\frac{2\pi \epsilon_{0}\epsilon_{r}}{\ln\left(\frac{D}{d}\right)} \end{array}$$

where *C* is the cable capacitance, *l* is the cable length, $\epsilon_{0}=8.854\times 10^{-12}$ F/m is the dielectric constant of free space, $\epsilon_{r}$ is the relative dielectric constant of the insulation, *D* is the inside diameter of the shield, and *d* is the outer diameter of the signal conductor.

Coaxial cable capacitance ranges from 30 pF/m to 100 pF/m depending on type [8]. Expected wire-end capacitance will be around 700 pF for a 7.4 m length, an approximate length expected from sensor at the pump to the electronics package. This value of capacitance is remarkably high and can pose a performance and stability problem for the inductive sensor.

**Figure 3:**
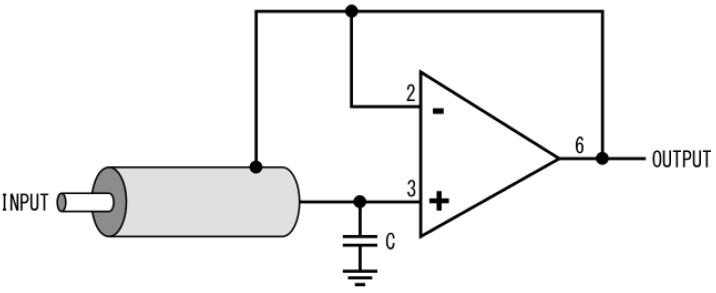
Coaxial cable guard circuit with unity gain buffer amplifier.

The standard approach to reducing cable capacitance is to use a guarded circuit [9,10]. Essentially, the shield is driven with a buffered input signal reducing the voltage difference between the shield and signal. Applied to coaxial cables, this reduces leakage and internal capacitance. Figure 3 shows a coaxial cable guard circuit from [9].

**Figure 4:**
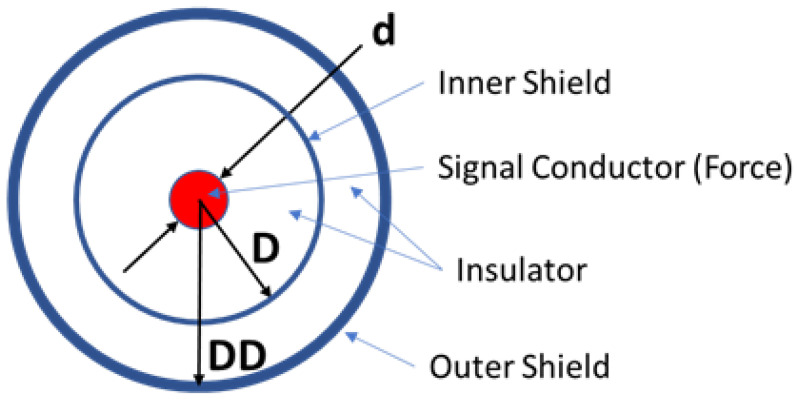
Cross section representation of a triaxial cable.

In the guard circuit, the coaxial shield is no longer grounded, which means that extraneous electrical and magnetic interference cause a voltage differential between the shield and central conductor. This means that the noise is no longer common mode, and cannot be rejected by the differential amplifier. Additionally, the amplifiers output impedance is not zero, which means that extraneous signals can be injected through this route. To address these limitations of coaxial cables, a triaxial cable with two independent shields and an inner conductor was chosen to transmit the sensor signal. The triaxial cable geometry is shown in Figure 4.

The triaxial cable’s inner shield can be driven like the guard circuit, but the cable also has another outermost shield that is ground connected to prevent the inner shield from receiving extraneous voltages and currents.

### Cable Insulation

For high temperature applications, it is impossible to use plastic insulated cables. Instead, mineral insulated cables are typically used. Most mineral insulated cables use compressed magnesium oxide (MgO) powder as the insulator. This oxide insulator withstands high temperatures and has long-term stability. It is also manufactured easily, owing to the powder’s compressibility. However, the dielectric constant of MgO is about 6.5 [11], which is a significant increase in capacitance compared to polyethylene insulation’s 2.3 [12]. Therefore, the capacitance value of a mineral insulated cable will be about 2.8 times that of an otherwise equivalent plastic insulated cable. This higher constant could result in over 1700 pF of capacitive loading from a 7.4 m span cable.

Aluminum oxide is also used as an insulator in mineral insulated cables, and it has a dielectric constant of about 9 [12], which will present as four times as much capacitance as plastic insulation. Coupled with the large physical separation between the sensor and the signal processing hardware, this makes reducing the apparent cable capacitance even more critical for high temperature applications if high bandwidth measurements are required.

## 2. Materials and Methods

A difficulty is encountered in driving the inner shield, which was not present in the guard circuit proposed in [9]. There exists a very high capacitance between the driven inner shield and the outermost shield. This high capacitance causes instability and oscillations in the operational amplifier driving the shield. This was solved by decoupling the unity-gain buffer amplifier from the inner shield and driving the shield with a separate driver amplifier. Still, the separate driver amplifier will have great difficulty with a high-capacitance load. Hence, a resistor–inductor compensator is placed in series between the driver amplifier and the inner shield to stabilize the guard circuit.

### 2.1. Simulation

**Figure 5:**
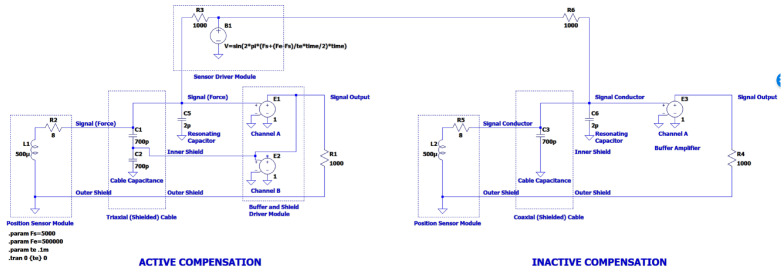
LTSpice simulation of the triaxial driver. The **left** side is performing the active compensation. The **right** side is a standard cable.

A model of the capacitance compensation system was first simulated using LTSpice to determine performance and stability. The model is shown in Figure 5. Two circuits are simulated simultaneously:

1. Active compensation;
2. Compensation off.

**Figure 6:**
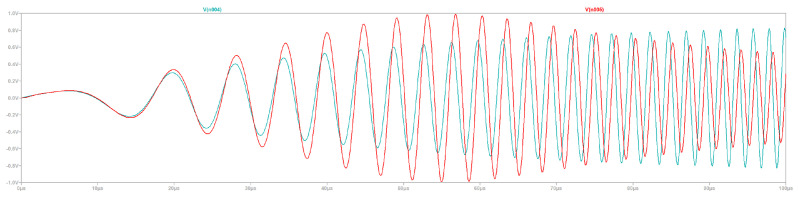
Frequency sweep plot from 5 kHz to 500 kHz with active (green) and inactive compensation (red).

The results given in Figure 6 compare the active and inactive case. Consistent response over the frequency range is demonstrated by the active compensation circuit. The physical experiments described below verify that the circuit functions as intended.

### 2.2. Experiment

The cable capacitance technique described above was tested using a custom eddy current sensor. The intended application of this sensor is for continuous position sensing at 750 °C with a 5 kHz bandwidth. A low temperature analog of the high temperature sensor was used to test the active capacitance compensation.

**Figure 7:**
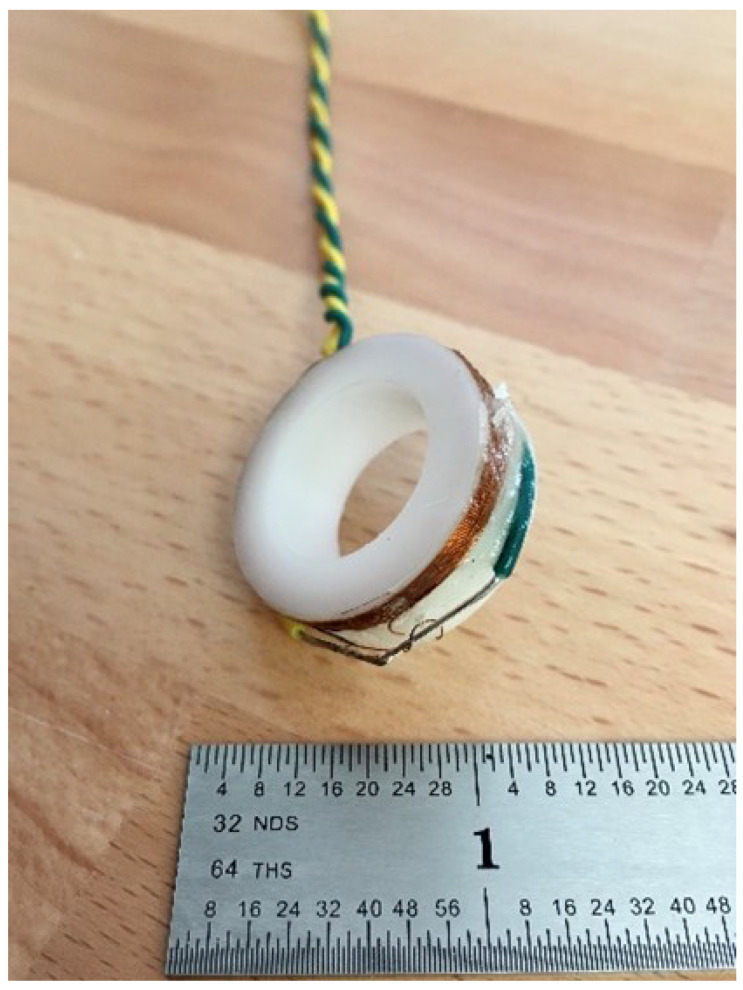
Coil design used for testing the capacitance reduction method.

The sensor is wound on a polyoxymethylene (Delrin) spool. The outer diameter is 28 mm and the inside diameter is 17.5 mm. The coil is 100 turns of 34 American Wire Gauge (AWG) enamel coated copper wire. The coil is shown in Figure 7.

**Figure 8:**
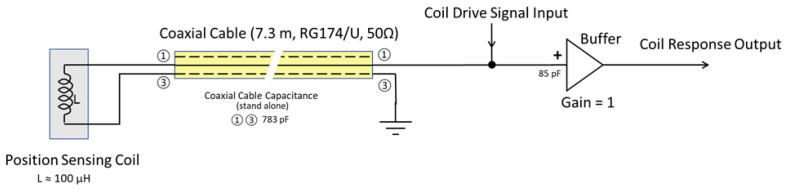
Coil sensor connection using coaxial cable.

**Figure 9:**
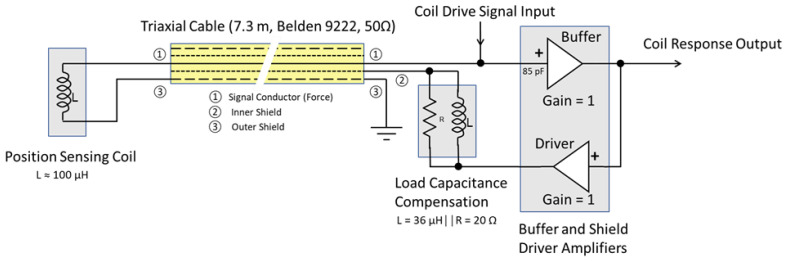
Coil sensor connection using triaxial cable with active capacitance compensation.

To test the active capacitance reduction, the active technique using a triaxial cable was compared to both coaxial and triaxial cable without active compensation. A schematic of the coaxial sensor connection is shown in Figure 8, and the triaxial sensor connection with active capacitance reduction is shown in Figure 9.

The coaxial cable tested was a 7.3 m section of regular RG174/U cable (see Table 1 for dimensions and construction). The triaxial cable tested was a 7.3 m section of Belden 9222 triaxial cable (see Table 2 for dimensions and construction).

**Figure 10:**
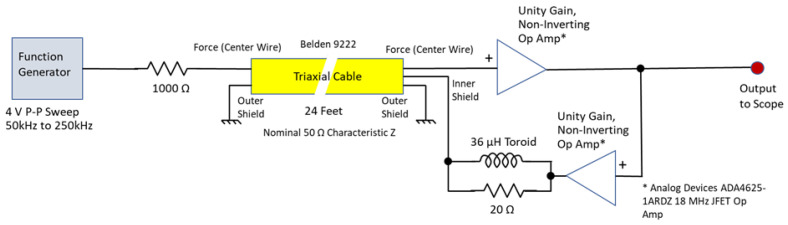
Active cable capacitance reduction test circuit.

Actual capacitance measurements were taken using a BK Precision model 880 LCR meter. The test circuit used to measure the apparent capacitance reduction is shown in Figure 10.

The circuit shown in Figure 9 shows how the circuit would be used in practice when connected to the sensor shown in Figure 7, where the circuit shown in Figure 10 is the circuit used to measure the reduction in cable capacitance.

**Figure 11:**
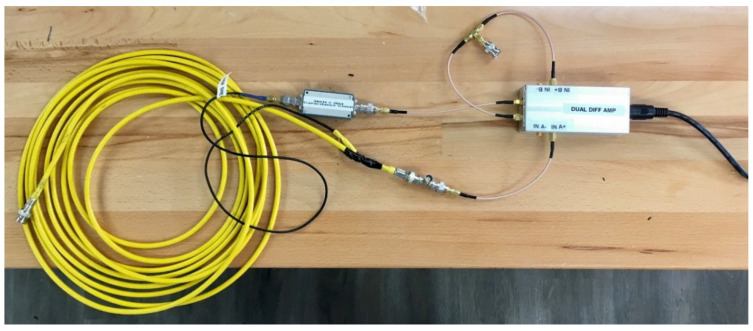
Physical hardware and 7.3 m of triaxial cable capacitance compensation system shown in Figure 10.

**Figure 12:**
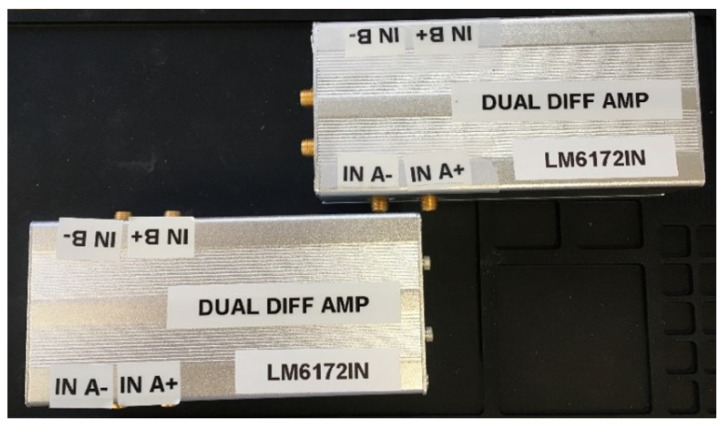
Active cable capacitance compensation amplifier prototypes.

The physical hardware for testing the active capacitance reduction is shown in Figure 11, and the prototype amplifiers are shown in Figure 12.

Note that the Analog Devices ADA4625 operational amplifiers, which were initially selected, did not have sufficient input voltage range to perform well over the sensor’s entire operating regime; therefore, Texas Instruments LM6172-2 dual high-speed operational amplifiers replaced them for the final prototype. The LM6172-2 integrated circuits have exceptionally high slew rate and unity-gain bandwidth (3000 V/µs, 100 MHz).

## 3. Results

The static capacitance values for the 7.3 m triaxial and coaxial cables are presented in Table 3. These cable capacitance values will decrease the sensor measurement bandwidth and signal-to-noise ratio. Note the exceedingly high capacitance between the inner shield and the outer shield. This high value results from large diameter of the inner shield. As mentioned above, although it does not directly affect the signal path, it presents a difficulty for the op amp driving the shield. A resistor and inductor in parallel are used to stabilize the operational amplifier driving the this large capacitive load.

The static capacitance measurements do not include the buffer amplifier input capacitances, which were about 85 pF. Table 4 shows the result of activating the buffer driven circuit in Figure 10.

The circuit reduces apparent capacitance between center signal conductor and outer shield (which serves as ground shield and signal return path) to about 14 pF. This dynamic reduction represents a 45X improvement. The active capacitance compensation removes all but about 2% of the 627 pF static capacitance of the triaxial cable, while still maintaining a grounded shield for mitigating against the effects of extraneous signals on the measurement by common mode rejection.

## 4. Discussion

The active cable capacitance reduction technique presented virtually eliminates the apparent cable capacitance as seen by the sensor. This permits high-bandwidth measurements with improved signal-to-noise ratios at the receiving end. While the specific application motivating this work is sensing in extreme environments such as ultra-high temperatures, it has applications beyond extreme environment sensing. For example, it could be used to create high-speed data pipelines for large data centers. It could also be used to create analog sensor networks over large geographical areas.

## Figures and Tables

**Table 1:** Construction of a regular RG174/U coaxial cable.

Layer	Material	Outer Diameter [mm]
Inner Conductor	Bare Copper Clad Steel	0.48
Insulation	Polyethylene	1.52
Shield Layer	Tinned Copper	1.93
Jacket	Polyvinyl Chloride	2.80

**Table 2:** Construction of a Belden 9222 triaxial cable.

Layer	Material	Outer Diameter [mm]
Inner Conductor	Tinned Copper	0.965
Insulation	Polyethylene	3.05
Inner Shield	Tinned Copper	3.61
Inner Jacket	Polyethylene	4.50
Outer Shield	Tinned Copper	5.05
Outer Jacket	Polyvinyl Chloride	6.12

**Table 3:** Static inter-electrode capacitance for triaxial and coaxial shielded cables.

Measurement Point ^1^	Cable Type	Cable Capacitance [pF]
➀➁	Triaxial	777
➀➂	Triaxial	627
➁➂	Triaxial	3170
➀➂	Coaxial	783

^1^ See Figure 8 and Figure 9.

**Table 4:** Measured cable capacitance as observed by sensor looking into the 7.3 m long Belden 9222 triaxial cable.

Measurement Point ^1^	Cable Type	Cable Capacitance [pF]	Percent Decrease
➀➂	Belden 9222 Triaxial	14	98

^1^ See Figure 8 and Figure 9.

## Data Availability

No new data were created or analyzed in this study. Data sharing is not applicable to this article.

## Abbreviations

The following abbreviations are used in this manuscript:

ARPA-E	Advanced Research Projects Agency-Energy
AWG	American Wire Gauge
DOE	Department of Energy
MSR	Molten Salt Reactor
NASA	National Aeronautical and Space Administration
